# Supplementary material for: Building the bioeconomy: A targeted assessment approach to identifying biobased technologies, challenges and opportunities
Source: Eng Biol. 2024 Feb 7;8(1):1–15. doi: 10.1049/enb2.12030 (PMC10959757; doi:10.1049/enb2.12030)
Supplement: Supplementary file 1 — Supporting Information S1 [file ENB2-8-1-s001.docx]

**Supplementary Materials (S)**

**S1.1 Overview**

This supplementary materials document provides additional details on the targeted assessment process.

**S1.2** **Bioeconomy technologies: long list generation**

An initial list of technologies that could contribute to bioeconomy development was formulated. Generating this long list was aided by a search for publications in the Web of Science (WoS) Core Collection. The search was targeted to the most recent period (2015-2020) due to the focus of the project on new and emerging technologies. Search terms included “Biobased”, “Bio-based”, “Biomaterial” or “Synthetic Biology”. Results were categorised into 25 WoS subject categories including Biotechnology Applied Microbiology, Biochemistry Molecular Biology, and Plant Science but excluding categories relating to medicine or medical applications that were outside of the scope of this work. Publications were then sorted within each of the remaining disciplines and ranked by citations. Manual review was undertaken of 10 high-ranking but diverse papers from each subject category. This was intended both to allow sufficient depth within a given subject category but to also allow breadth across disciplines. Following this, Google Scholar was used, alongside online patent, publication, and media resources, to identify other non-medical bioeconomy-relevant technologies. The aim of this approach was not to exhaustively identify every possible technology, but rather to develop a sample long list that was sufficiently diverse, from different disciplines and feedstocks, to encompass a relatively broad section of the landscape of potential bioeconomy technologies. Concise profiles of potential technologies were developed by the author research team based on review of descriptions of the technology under development and available documentation (through publications, patents, or websites). There was a focus on technologies that were generic (i.e., not highly company specific) and either entering or relatively close to market – i.e., which could be implemented within, as a maximum, the next 5-10 years. A total of 50 potential bioeconomy technologies emerged (see Table S1).

**Table S1: Long list bioeconomy technologies and bio-based production requirements fulfilment**

| No. | Bioeconomy technology | Biobased selection requirements | | | | | Biobased short List |
| --- | --- | --- | --- | --- | --- | --- | --- |
|  |  | Biobased | UK Applicable | Economic | Environmental | Societal |  |
| 1 | Graphene biocomposites | **⭘** |  |  |  |  |  |
| 2 | **Biomethane** | **🞈** | **🞈** | **🞈** | **🞈** | **🞈** | **🗹** |
| 3 | Hydrogen Fuel Cells | **⭘** |  |  |  |  |  |
| 4 | Ocean Thermal Energy | **⭘** |  |  |  |  |  |
| 5 | Shallow Geothermal | **⭘** |  |  |  |  |  |
| 6 | Electric Transport Network | **⭘** |  |  |  |  |  |
| 7 | Carbon Capture and Storage | **⭘** |  |  |  |  |  |
| 8 | **GMO Biomass** | **🞈** | **🞈** | **🞈** | **🞈** | **⭘** | **🗹** |
| 9 | Guayule Rubber | **🞈** | **⭘** |  |  |  |  |
| 10 | **Microfibrilated Cellulose (MFC)/Nanocellulose** | **🞈** | **🞈** | **🞈** | **🞈** | **🞈** | **🗹** |
| 11 | **Lignin Biocomposites reinforced with Plant Fibres** | **🞈** | **🞈** | **🞈** | **🞈** | **🞈** | **🗹** |
| 12 | **Thermoplastic Biopolymers reinforced with Plant Fibres** | **🞈** | **🞈** | **🞈** | **🞈** | **🞈** | **🗹** |
| 13 | **Plant Fibres reinforced with bioresin pre-pregs** | **🞈** | **🞈** | **🞈** | **🞈** | **🞈** | **🗹** |
| 14 | **Self-binging Composite non-woven Plant Fibres** | **🞈** | **🞈** | **🞈** | **🞈** | **🞈** | **🗹** |
| 15 | **Biolubricants** | **🞈** | **🞈** | **🞈** | **🞈** | **🞈** | **🗹** |
| 16 | **PHAs from Urban Waste** | **🞈** | **🞈** | **🞈** | **🞈** | **🞈** | **🗹** |
| 17 | Biobased Polyamide-12 | **🞈** | **🞈** | **🞈** | **⭘** |  |  |
| 18 | **Lignin-based Carbon Nanofibres** | **🞈** | **🞈** | **🞈** | **🞈** | **🞈** | **🗹** |
| 19 | Bio BTX aromatics | **🞈** | **🞈** | **🞈** | **⭘** |  |  |
| 20 | **Lignin-based Phenolic resins** | **🞈** | **🞈** | **🞈** | **🞈** | **🞈** | **🗹** |
| 21 | **Lignin Bio-oil** | **🞈** | **🞈** | **🞈** | **🞈** | **🞈** | **🗹** |
| 22 | **High Purity Lignin** | **🞈** | **🞈** | **🞈** | **🞈** | **🞈** | **🗹** |
| 23 | Bio-based Phenol and Alkyl Phenols | **🞈** | **🞈** | **🞈** | **⭘** |  |  |
| 24 | Bioethanol | **🞈** | **🞈** | **🞈** | **⭘** |  |  |
| 25 | Biopropane | **🞈** | **🞈** | **🞈** | **⭘** |  |  |
| 26 | GMO Cotton |  | **🞈** | **⭘** | **⭘** |  |  |
| 27 | Static Electricity Catalysis | **⭘** |  |  |  |  |  |
| 28 | Metallic Hydrogen | **⭘** |  |  |  |  |  |
| 29 | **Limonene-based engineering polymers** | **🞈** | **🞈** | **🞈** | **🞈** | **🞈** | **🗹** |
| 30 | Bacterial Biosurfactants | **🞈** | **🞈** | **⭘** |  |  |  |
| 31 | **Biotechnical Chitosan** | **🞈** | **🞈** | **🞈** | **🞈** | **🞈** | **🗹** |
| 32 | Volatile Fatty Acid Mixtures | **🞈** | **🞈** | **🞈** | **⭘** |  |  |
| 33 | SKH Metallic Trees | **⭘** |  |  |  |  |  |
| 34 | Space Solar Power Stations | **⭘** |  |  |  |  |  |
| 35 | Fatty Acids as Phase Change Materials | **🞈** | **🞈** | **⭘** |  |  |  |
| 36 | **Seaweed Technology** | **🞈** | **🞈** | **🞈** | **🞈** | **🞈** | **🗹** |
| 37 | Reusing Nuclear Waste | **⭘** |  |  |  |  |  |
| 38 | Bio-based Jet Fuels | **🞈** | **🞈** | **⭘** |  |  |  |
| 39 | Wave and Tidal Power | **⭘** |  |  |  |  |  |
| 40 | **Lactic Acid-based Bioplastics** | **🞈** | **🞈** | **🞈** | **🞈** | **🞈** | **🗹** |
| 41 | Smog-free towers | **⭘** |  |  |  |  |  |
| 42 | High-pressure Diamond Synthesis | **⭘** |  |  |  |  |  |
| 43 | Solar Sea Cleaners | **⭘** |  |  |  |  |  |
| 44 | **Alternative Fabrics (e.g., S Café)** | **🞈** | **🞈** | **🞈** | **🞈** | **🞈** | **🗹** |
| 45 | Cloudfisher | **⭘** |  |  |  |  |  |
| 46 | Grid-scale Renewable Storage (Molta – Google X) | **⭘** |  |  |  |  |  |
| 47 | Synthetic Omega-3 | **⭘** |  |  |  |  |  |
| 48 | Central Enzyme Bank | **⭘** |  |  |  |  |  |
| 49 | Feed Additions for livestock (Methane reduction) | **🞈** | **🞈** | **⭘** |  |  |  |
| 50 | Biodichloromethane | **🞈** | **🞈** | **🞈** | **⭘** |  |  |

Source: Evaluation of long-list of potential bioeconomy technologies based on author review of literature and workshop/seminar feedback. Requirements key: **🞈** performed; **⭘** partially performed; **⭘** not performed; **🗹** advanced to bio-based short list.

**S1.3 Biobased production technologies: short-list generation**

Technologies from the long list were further assessed on their ability to fulfil the following requirements: (1) Bio-based production technologies using the definition of “biobased” in the scope of the project (Non-food products derived from renewable biomass); (2) Nationally (UK) applicable – utilisation of native feedstock, resources, and expertise; (3) Economically beneficial – either by reducing dependence on imports, bolstering a particular market, or providing new goods or functionalities; (4) Socially positive – allowing local management of resources, job creation or other societal gains; and (5) Environmentally benign (or the potential to be so) – considering greenhouse gas (GHG) emissions toxicity, resource depletion, habitat destruction, water use, etc. In addition to author review, the list in progress was presented to experts in biotechnology, engineering biology, and science and technology policy (via workshops or seminars at The Future Biomanufacturing Research Hub; the Manchester Institute of Innovation Research; and the Manchester Synthetic Biology Research Center, and with members of the Institute for Food and Resource Economics at the University of Bonn). Of the original long list, 18 technologies were determined to fulfil the requirements criteria established and were included on the short-list of promising biobased production technologies. (Table S1).

**S1.4 Focus Group**

For the focus group, six technologies were selected from the short list as ‘exemplary technologies’ that represented a range of different feedstocks, low- and high-value products, and a diverse range of primary end uses. The technologies were:

1. Biomethane
2. High Purity Lignin
3. Microfibrilated Cellulose (MFC)/Nanocellulose
4. PHAs from fatty acids and urban waste
5. Seaweed technology
6. Bioethanol

Stakeholders represented industry leaders, academics, science platforms, think-tanks, government, policy, and feedstock providers, comprising 10 attendees plus two research team members. The session was hosted via Zoom in January 2021. Following an introduction of the project and explanation of its aims and selection criteria for bio-based technologies, focus group participants were split into two groups, each focussing on three of the exemplary technologies (**Group A:** Biomethane, MFC/nanocellulose, PHAs from urban waste; **Group B:** Bioethanol, High Purity Lignin, Seaweed Technology), and asked to answer three questions:

1. What are the specific challenges you see relevant to each of these specific technologies? E.g., technology/ management/ regulation, etc.
2. What are the main governance/ funding/ regulatory roadblocks you consider to be the most important and how can these be overcome?
3. Are there any other technologies you think should be included as exemplary case studies based on the UK’s native feedstocks/resources/expertise or policy drives?

Following discussion between stakeholders within the breakout groups, each group was asked to present their conclusions to the entire focus group, allowing further discussion of all technologies. After the focus group, feedback was provided to all participants, allowing them to provide additional insight or resources, and generate further discussion.

**S1.5 Case Studies**

Case studies were created for lignin, cellulose, and seaweed using online resources, peer-reviewed publications, patents, and direct communication with field experts and industry. The case studies focussed on the following probes (Figure S1):

1. Functionality: Technical specification, novel functions, and comparison to the status quo.
2. Sustainability: Source of feedstock, ability of UK to provide / import, and sustainability/environmental issues of production and consumption.
3. Development: Current use, and alternative and developing methods of utilisation, extraction, and valorisation.
4. Market and Customers: Affected markets and customers, current and potential scale / growth / locations of the market, and potential market competitors.
5. Scaling: Potential for and requirements of scalability of the extraction/ valorisation process, and examples of technologies in development.
6. Industry/ Investment/ Partners: Current industry structure, potential for redistributed (small-scale, decentralised) production, and capital requirements.
7. Governance: Regulatory aspects, public acceptance and possible opposition to this feedstock / technology.

Figure S1: Case study probes
